# Distinct pleiotropic effects of direct oral anticoagulants on cultured endothelial cells: a comprehensive review

**DOI:** 10.3389/fphar.2023.1244098

**Published:** 2023-09-29

**Authors:** Natalia Atzemian, Dimitra Kareli, Georgia Ragia, Vangelis G. Manolopoulos

**Affiliations:** ^1^ Laboratory of Pharmacology, Medical School, Democritus University of Thrace, Alexandroupolis, Greece; ^2^ Individualised Medicine and Pharmacological Research Solutions Center (IMPReS), Alexandroupolis, Greece; ^3^ Clinical Pharmacology Unit, Academic General Hospital of Alexandroupolis, Alexandroupolis, Greece

**Keywords:** DOACs - direct oral anticoagulants, endothelial cells, pleiotropic effects, DOACs heterogeneity, dabigatran, rivaroxaban, apixaban, edoxaban

## Abstract

Direct Oral Anticoagulants (DOACs) have simplified the treatment of thromboembolic disease. In addition to their established anticoagulant effects, there are indications from clinical and preclinical studies that DOACs exhibit also non-anticoagulant actions, such as anti-inflammatory and anti-oxidant actions, advocating overall cardiovascular protection. In the present study, we provide a comprehensive overview of the existing knowledge on the pleiotropic effects of DOACs on endothelial cells (ECs) *in vitro* and their underlying mechanisms, while also identifying potential differences among DOACs. DOACs exhibit pleiotropic actions on ECs, such as anti-inflammatory, anti-atherosclerotic, and anti-fibrotic effects, as well as preservation of endothelial integrity. These effects appear to be mediated through inhibition of the proteinase-activated receptor signaling pathway. Furthermore, we discuss the potential differences among the four drugs in this class. Further research is needed to fully understand the pleiotropic effects of DOACs on ECs, their underlying mechanisms, as well as the heterogeneity between various DOACs. Such studies can pave the way for identifying biomarkers that can help personalize pharmacotherapy with this valuable class of drugs.

## 1 Introduction

Anticoagulant drugs constitute the mainstay of thrombus prophylaxis in individuals at high risk for thromboembolic events, most notably venous thromboembolism (VTE) and stroke in atrial fibrillation (AF) (Graf and Tsakiris, 2012). Vitamin K antagonists (VKAs) have been the standard anticoagulation treatment for over 60 years, providing significant therapeutic benefit and saving millions of lives. However, VKAs present several clinical drawbacks that necessitated the development of direct oral anticoagulants (DOACs). DOACs constitute a major innovation in the field, and since their introduction in clinical practice in 2008, they have gradually dominated over VKAs and have revolutionized anticoagulation treatment (Pirmohamed, 2006; Schwarb and Tsakiris, 2016; Lippi et al., 2017). Unlike VKAs, which target a gamut of clotting factors, DOACs directly inhibit specific factors, such as thrombin (dabigatran) or activated factor X (FXa) (rivaroxaban, edoxaban, and apixaban) within the coagulation cascade. DOACs provide a better safety and efficacy profile since they are short-acting, rapid agents, that reversibly bind to their targets (Graf and Tsakiris, 2012). Their main advantages over VKAs include simpler clinical use, fewer drug-drug and food-drug interactions, no need for routine monitoring, and a lower bleeding risk (Granger et al., 2011; Patel et al., 2011; Hart et al., 2012).

It is often the case that medications, including those for cardiovascular diseases (CVDs), turn out to have beneficial actions beyond those they were developed for, so-called “pleiotropic effects”. Statins, which were introduced in the early '90s to combat hypercholesterolemia, are a prominent example. Since then, their impact has surpassed cholesterol reduction, offering a plethora of diverse cardiovascular protective actions, including support of endothelial integrity and function (Marzilli, 2010).

However, whether DOACs have such pleiotropic effects remains a topic of ongoing investigation. It is conceivable that their actions can potentially extend beyond their conventional role in anticoagulation by controlling the activity of two serine proteases; factor Xa (FXa) and thrombin. It is well established that these proteases mediate several (patho)physiological processes such as inflammation, atherothrombosis and angiogenesis (Danckwardt et al., 2013; Esmon, 2014) by triggering the activation of proteinase-activated receptors (PARs). PARs are a family of G protein-coupled receptors located on the cell surface, also known as thrombin receptors (coagulation factor II thrombin receptor, F2R), and orchestrate a multitude of cellular responses (Coughlin, 2000; Borensztajn et al., 2009). The ability of DOACs to regulate PARs responses by inhibiting thrombin and FXa opens new insights into their actions beyond anticoagulation.

Endothelial cells (ECs) line the luminal surface of blood vessels, which consists of the direct contact of flowing blood with the vessels. They are guardians of vascular integrity by regulating blood pressure and secreting critical molecules such as nitric oxide and prostacyclin (Mitchell et al., 2008). Endothelial dysfunction due to mechanical damage or chronic inflammation leads to CVDs, such as atherosclerosis, thrombotic events, and stroke (Wu and Thiagarajan, 1996; Rajendran et al., 2013). Cultured ECs constitute a well-established *in vitro* model of the vasculature and since the '70s they have been used in a myriad of studies, providing valuable mechanistic insight on the full spectrum of CVDs. Considering that ECs express all four PARs, a potential link emerges between dysregulation of PAR signaling and ECs dysfunction, which may be modulated by DOACs (Grimsey and Trejo, 2016; Seki et al., 2017). Hence, it can be hypothesized that DOACs have the potential to modulate endothelial function and induce pleiotropic effects by regulating the activation of PARs in ECs.

This comprehensive review aims to present the current state of knowledge regarding the effects of DOACs in ECs *in vitro*. We further discuss the mechanisms by which DOACs exert their pleiotropic actions, as they can be extrapolated from these *in vitro* studies. This could be beneficial in identifying novel actions of DOACs and better understanding the entire range of their impact. An additional aim of this study is to identify potential differences between the four agents in this class. Currently, the choice of DOACs for each patient is based mostly in clinical criteria and clinical experience. Uncovering potential discriminatory factors could help in personalizing DOACs drug therapy.

## 2 Methods

In March 2023, two reviewers independently screened the titles and abstracts of the retrieved studies through a literature search of the National Library of Medicine (PubMed). The literature search was conducted for the four commercially available DOACs (apixaban, rivaroxaban, dabigatran, and edoxaban). The keywords/strings used were (endothelial) AND (cells) AND (rivaroxaban) OR (apixaban) OR (dabigatran) OR (edoxaban) OR (DOACs) OR (NOACs) OR ((direct) AND (oral) AND (anticoagulants)) OR ((new) AND (oral) AND (anticoagulants)) OR ((direct) AND (thrombin) AND (inhibitor)) OR ((direct) AND (factor) AND (Xa) AND (inhibitor)) OR (in) AND (vitro) AND (studies). There was no publication or language limit in the initial search, and the search engine displayed results from 2008 until the present. Only studies in human ECs were included. Duplicates were removed. We only included papers published in English. Full-text manuscripts were retrieved and reviewed.

## 3 Results

**FIGURE 1:**
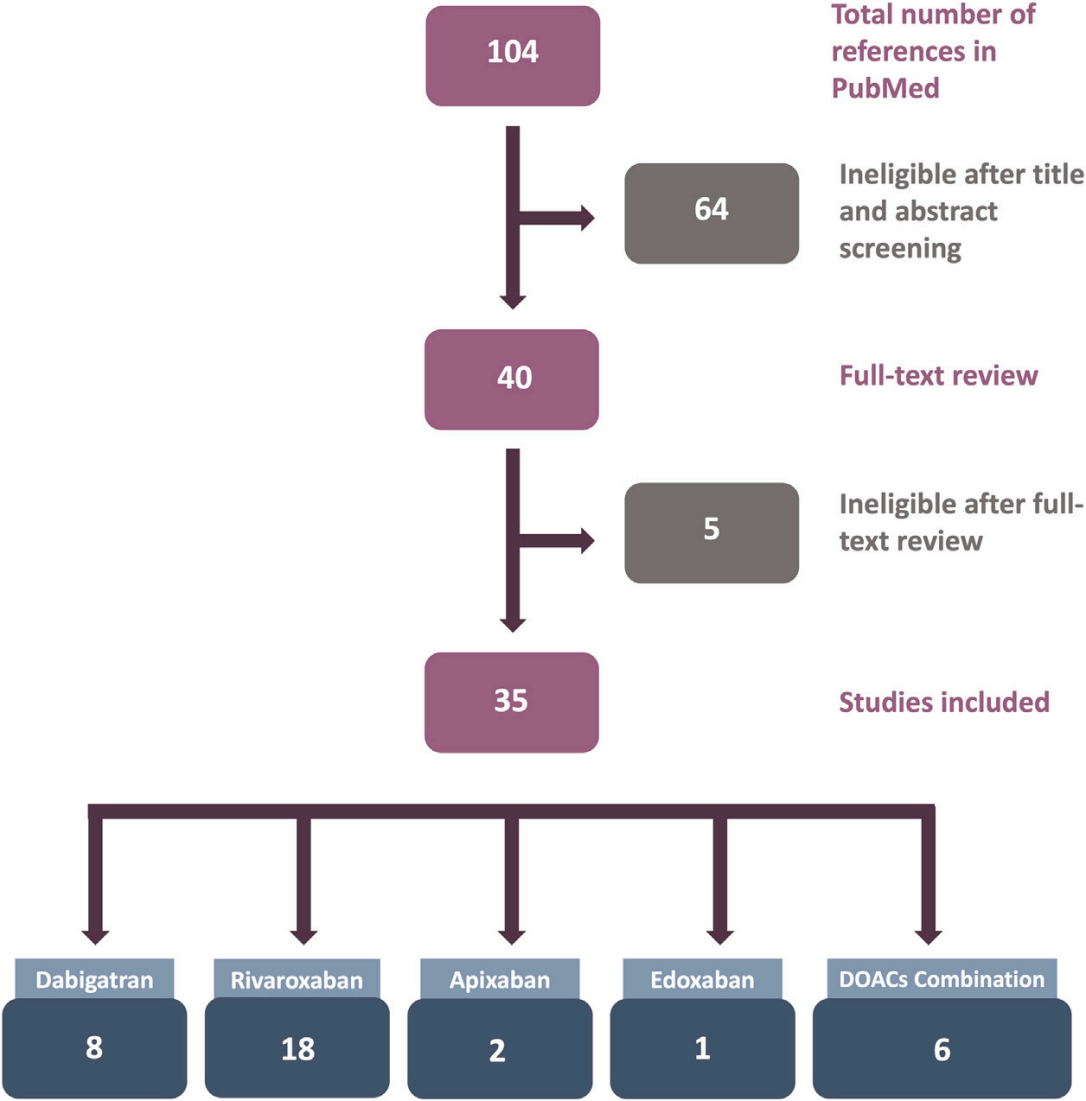
Flowchart of the results of the systematic review literature search. Only studies utilizing human endothelial cells (ECs) treated with direct oral anticoagulants (DOACs) in *in vitro* experiments were included.

The number of studies reviewed in this article is shown in Figure 1. After an initial search in PubMed, 104 articles were retrieved. After title and abstract screening, 64 articles were considered ineligible. For the remaining 40 publications, the full texts were reviewed for eligibility, and finally, 35 articles were included in this review.

## 4 *In vitro* experiments using DOACs on ECs

### 4.1 Dabigatran

Dabigatran is the first DOAC that was approved by the Food and Drug Administration (FDA) in 2008 and revolutionized the antithrombotic treatment. It is prescribed for the prevention of thromboembolic events in high-risk patients, such as patients with non-valvular AF (NV-AF), pulmonary embolism (PE), and VTE, while it is the first anticoagulant approved for VTE treatment in children (van Ryn et al., 2013; Kuehn, 2021). Dabigatran is a pro-drug, dabigatran etexilate, and it is 80% renally excreted. Since the introduction of dabigatran to the market, eight studies have examined its effects on human ECs. A summary of the pleiotropic effects of dabigatran on ECs *in vitro* is shown in Table 1.

**TABLE 1:** Overview of the pleiotropic effects of dabigatran in endothelial cells (ECs) *in vitro*.

Endothelial cell type	Dabigatran dose	Stimulation factor	Cellular effects of dabigatran	Biological/Pleiotropic effects	Reference
**HUVECs**	160, 800 nM	25-hydroxycholesterol	↓ Endothelial permeability, *VEGFA* expression	Stabilizes endothelial integrity	Gorzelak-Pabiś et al. (2022a)
			↑ CDH5 surface expression		
			↓ *ICAM1, IL-33, CCL2, TNF*	Anti-inflammatory effects	
**SECs**	500 nM	Ischemia	↑ PECAM1, THBD expression	Protection from ischemia and vascular integrity protection	Noguchi et al. (2021)
			↓ LDH Cytotoxicity, HMGB-1 expression		
**HLMVECs**	50 nM	IL-1β + thrombin-stimulated platelet releasate	↓ Platelet endothelial barrier-supportive function	Inhibition of platelet protection on pulmonary endothelium	Smeda et al. (2022)
**HUVECs**	10, 30, 100 nM	Thrombin (10 nM)	↓ *CXCC1, TWIST1* expression	Anti-angiogenic effects and anti-tumor effects	Vianello et al. (2016)
			↓ Tube formation		
**HUVECs**	100, 300 nM	Thrombin (10 nM)	↓ Endothelial permeability, Actin Stress Fiber Formation, MLC phosphorylation, Rho A GTPase activation	BBB integrity protection	Choi et al. (2018)
**HBMVECs**	250 nM	Glucose	↓ TNF, IL-6, MMP2, MMP9, NOS2, NOX4 expression	Glucose-induced neurodegeneration protection	Vittal Rao et al. (2021)
		Thrombin			
**HBEC-5i ECs + Human Astrocytes**	500 nM	Intermittent hypoxia + Thrombin (10–100 nM)	↓ Endothelial permeability, PAR-1/PAR-3 cleavage, ROS generation, HIF1A expression	Enhance endothelial barrier integrity	Zolotoff et al. (2022)
			↑ TJP1 expression		
**EA.hy926**	100, 300 nM	Thrombin (10 nM)	↓ PAR-1 cleavage, activation, internalization, and b-arrestin recruitment	PAR-1 signaling effects	Chen et al. (2015)
	10 μM		↑ PAR-1 surface expression, permeability		

HUVECs, Human Umbilical Vein Endothelial Cells; SECs, Sinusoidal Endothelial Cells; HLMVECs, Human Lung Microvascular Endothelial Cells; HBMVECs, Human Brain Microvascular Endothelial Cells; HBEC-5i, Human Brain Endothelial Cells-5 immortalized; EA.hy926 cells, Immortalized human umbilical vein endothelial cell line; VEGFA, Vascular Endothelial Growth Factor A; CDH5, Cadherin 5; ICAM1, Intercellular Adhesion Molecule-1; IL-33, Interleukin 33; CCL2, C-C Motif Chemokine Ligand 2; TNF, Tumor Necrosis Factor; PECAM1, Platelet Endothelial Cell Adhesion Molecule 1; THBD, Thrombomodulin; LDH, Lactate Dehydrogenase; HMGB-1, High Mobility Group Box 1; CXCC1, C-X-C Motif Chemokine 1; TWIST1, TWIST Family BHLH Transcription Factor 1; MLC, Myosin Light Chain; MMP2, Matrix Metalloproteinase-2; NOS2, Nitric Oxide Synthase 2; NOX4, NADPH Oxidase 4; PAR, Proteinase-Activated Receptor; ROS, Reactive Oxygen Species; HIF1A, Hypoxia Inducible Factor 1 Alpha; TJP1, Tight Junction Protein 1; BBB, Blood-Brain Barrier.

Atherosclerosis can be characterized as a chronic inflammatory disease of the vasculature, while there is a connection between atherosclerosis and disturbed hemostasis. In this notion, Gorzelak-Pabiś et al. (2022a) investigated the effects of dabigatran in an *in vitro* model of chronic atherosclerosis using 25-hydroxycholesterol (25-OHC)-induced human umbilical vein endothelial cells (HUVECs). Dabigatran reversed all oxysterol-induced effects. It enhanced endothelial integrity that had been damaged by 25-OHC. This was mediated by lessening mRNA expression of intercellular adhesion molecule 1 (*ICAM1*) and vascular endothelial growth factor A (*VEGFA*). Dabigatran was also able to stimulate the surface expression of cadherin 5 (CDH5, known also as VE-Cadherin) that had been reduced by 25-OHC and concurrently inhibited the transcription of 25-OHC-induced proinflammatory cytokines and chemokines interleukin 33 (*IL-33*), tumor necrosis factor (*TNF*), and C-C motif chemokine ligand 2 (*CCL2*). These results strongly suggest that dabigatran possesses anti-inflammatory effects and also enhances endothelial integrity (Gorzelak-Pabiś et al., 2022a).

Another study by Noguchi et al. (2021) was performed to mimic the damage to ECs caused by liver transplantation. They used, among others, sinusoidal ECs (SECs) to examine the effects of dabigatran in an *in vitro* hypoxia-reoxygenation (H-R) model. They found that dabigatran increased the protein expression of platelet and endothelial cell adhesion molecule 1 (PECAM1) and decreased high-mobility group box-1 (HMGB-1) secretion and lactate dehydrogenase (LDH) cytotoxicity levels in an H-R model. Dabigatran induced thrombomodulin (THBD) expression in cell lysates in both non-ischemic and H-R models, whereas in the H-R model, THBD levels in the supernatant of SECs pre-treated with dabigatran decreased. These findings suggest that dabigatran holds cytoprotective properties against hepatic ischemia and maintains vascular integrity, thereby preventing hepatic transplant rejection (Noguchi et al., 2021).

Two studies have been conducted to evaluate the effects of dabigatran on tumor growth and metastasis. In the study of Smeda et al. (2022), the impact of dabigatran on the pulmonary endothelial barrier and in metastasis spread was assessed both *in vivo* and *in vitro*. In the *in vitro* experiments, they used an inflammation model of human lung microvascular endothelial cell (HLMVEC) cultures. HLMVECs were stimulated by thrombin-activated platelet releasate, and, subsequently, their electrical resistance was measured throughout the induction process in both the presence and absence of IL-1β and dabigatran. These results revealed that dabigatran adequately abolished *in vitro* thrombin-activated platelet releasates’ ability to protect the pulmonary endothelial barrier against inflammatory stimuli (Smeda et al., 2022).

In the second study, the effect of dabigatran on tumor growth was evaluated. Among tumor cell lines, the team of Vianello et al. (2016) used also HUVECs to test the potential effects of thrombin and dabigatran in angiogenesis. HUVECs treatment with thrombin increased the mRNA expression of the angiogenetic proteins C-X-C motif chemokine ligand 1 (*CXCL1*, also known as GRO-α) and twist family bHLH transcription factor 1 (*TWIST1*), an effect that was restored by dabigatran. In tube formation assays in HUVECs, they also observed that thrombin alone stimulated tube formation, whereas the co-administration of thrombin and dabigatran reversed this effect. The ability of dabigatran to counteract thrombin-induced angiogenesis highlights its possible negative contribution to the vascularization of cancer metastasis (Vianello et al., 2016).

Endothelial integrity is a crucial factor in preserving the blood-brain barrier (BBB) from damage, potentially leading to intracerebral hemorrhage. From this perspective, the team of Choi et al. (2018) evaluated the effect of dabigatran on the endothelial barrier in thrombin-induced HUVECs. To assess endothelial permeability, they examined the monolayer of HUVECs by measuring the trans-endothelial electrical resistance (TEER), testing the morphological changes in the actin cytoskeleton, detecting myosin light chain (MLC) phosphorylation, and assessing the activity of Rho A GTPase. Dabigatran suppressed thrombin-induced effects on permeability, protecting and stabilizing endothelial barrier integrity, and elucidating a part of the underlying molecular mechanism (Choi et al., 2018).

There is a close connection between diabetes and neurodegeneration, as hyperglycemia can lead to neuroinflammation and microvascular dysfunction in the brain. To study the glucose-induced brain microvascular endothelial damage, Vittal Rao et al. (2021) treated human brain microvascular ECs (HBMVECs) with glucose, thrombin, dabigatran, and inhibitors of PAR-1, p38 mitogen-activated protein kinases (MAPK), matrix metallopeptidase 2 (MMP2), or MMP9. Glucose induced the expression of inflammatory proteins TNF, IL-6, MMP2, and MMP9, and of oxidative stress proteins nitric oxide synthase 2 (NOS2) and NADPH oxidase 4 (NOX4), which were attenuated by dabigatran. The addition of thrombin to HBMVECs resulted in overexpression of TNF, IL-6, p38, cAMP responsive element binding protein 1 (CREB1), MMP2, MMP9, NOS2, and NOX4, while the addition of dabigatran reversed this effect. These results show that dabigatran is efficient in neutralizing the effects of thrombin signaling through PAR-1 inhibition in damaged brain ECs by glucose and, thus, potentially protecting them from neurodegeneration (Vittal Rao et al., 2021).

Patients with obstructive sleep apnea can present endothelial damage in BBB due to chronic intermittent hypoxia. This event has been evaluated in the recent research work by Zolotoff et al. (2022) They used a co-culture of human astrocytes cell line and human brain endothelial cells (HBEC-5i) to assess the effects of intermittent hypoxia with thrombin on the BBB. Under intermittent hypoxic conditions, low doses of thrombin exerted a positive effect on the BBB, with significant PAR-3 cleavage. In contrast, high concentrations of thrombin harmed BBB permeability, by activating reactive oxygen species (ROS) and PAR-1 and led to the upregulation of the expression of hypoxia inducible factor 1 subunit alpha (HIF1A) and downregulation of tight junction protein 1 (TJP1). When brain ECs were pretreated with dabigatran, both of the aforementioned beneficial and harmful thrombin effects were inhibited. These outcomes illustrate the biphasic effects of dabigatran in high and low exposure to thrombin; specifically, dabigatran exhibits beneficial effects on endothelial permeability at high concentrations of thrombin (Zolotoff et al., 2022).

From a different perspective from the previous works, Chen et al. (2015) examined whether thrombin-bounded dabigatran is able to attach to PAR-1 and can thus affect the endothelial barrier permeability using human umbilical vein endothelial-derived EA.hy926 cells. They observed that endothelial permeability increased when PAR-1-agonist-treated cells were exposed to thrombin-bound dabigatran compared with dabigatran alone. They reported that, at normal levels, dabigatran inhibits the catalytic site of thrombin and its capacity to bind with substrates such as PAR-1 and may control their functions. However, prolonged exposure to catalytically inactive thrombin, treated with supratherapeutic concentrations of dabigatran, resulted in increased surface expression of PAR-1 and enhanced signaling (Chen et al., 2015).

### 4.2 Rivaroxaban

Rivaroxaban is the first direct FXa inhibitor approved by FDA in 2011 for stroke prevention in NV-AF patients and VTE and PE treatment. It has the shortest half-life of all DOACs (Yates, 2014) and it inhibits FXa by forming two hydrogen bonds with the amino acid Gly219 (Yang et al., 2017). Rivaroxaban is, by far, the DOAC that has been studied most extensively *in vitro*. Table 2 summarizes the *in vitro* effects of rivaroxaban on ECs.

**TABLE 2:** Overview of the pleiotropic effects of rivaroxaban in endothelial cells (ECs) *in vitro*.

Endothelial cell type	Rivaroxaban dose	Stimulation factor	Cellular effects of rivaroxaban	Biological/Pleiotropic effects	Reference
**EA.hy926**	500 nM	FXa	↑ Tube formation	Angiogenic effects	Lange et al. (2014)
**HUVECs**	10 μM	FXa (10 nM)	↑ Proliferation	Proliferative action	Sanada et al. (2016)
			↑ Tubular length	Angiogenic effects	
			↓*CDKN2A, CDKN1C, EGR1, IGFBP-5, IL-1*β*, IL-6, CCL2, ICAM1 expression*	Anti-inflammatory effects	
**HUVECs**	500 nM	FXa (50 and 100 nM)	↓ *ICAM1, CCL2, IL-8 expression*	Anti-inflammatory effects	Seki et al. (2017)
			↓ CCL2 secretion		
**HaECs**	1 μM	FXa (50 nM)	↓ *IL-1*β*, IL-6, IL-8, CCL2, ICAM1, VCAM1, MMP2* expression	Anti-inflammatory effects	Ding et al. (2021)
			↓ Monocytes adhesion to ECs		
**HUVECs**	50 nM	FXa (9 nM)	↑ Proliferation	Proliferative action	Álvarez et al. (2018)
			↑ Migration	Angiogenic effects	
			↓ *EDN2, SELE, CCL5, VCAM1, MMP2*, u-PA expression, platelet adhesion	Anti-inflammatory effects	
			↑ u-PA activity		
**HCAECs and HDBECs**	137.9, 1379 nM	FXa (10 nM)	↓ Endothelial permeability	Stabilizes endothelial integrity	Benelhaj et al. (2019)
**HUVECs**	30 nM	AGEs + 3% citrated-plasma	↓ *MOK, CCL2, ICAM* expression	Anti-inflammatory effects	Ishibashi et al. (2014)
			↓ ROS generation, THP-1 cell adhesion	Anti-oxidant effects	
**HUVECs**	30 nM + Ang II	AGEs	↑*TFPI* expression and activity	Angiotensin II-mediated anticoagulant effects	Yang et al. (2017)
**HUVECs**	50, 500 nM	High glucose (22 mM)	↓ Senescence, p53, p16 expression	Anti-senescence effects	Maeda et al. (2019)
			↑ Telomerase activity and telomere length		
			↑ NOx, NOS3 expression	Anti-atherosclerotic effects	
			↓ ROS generation, p22^phox^, ICAM1, VCAM1, PAR-1 expression		
**HCAECs**	50 nM + Aspirin	D-Glucose (30 mM)	↑ PRKN, PINK1 expression	Promote mitophagy	Zekri-Nechar et al. (2022a)
			↓ ROS generation	Anti-oxidant effects	
**EPCs**	5, 10, 20 μM	High glucose (20 mM)	↑ Proliferation, migration, tube formation, NOS3, AKT1, p-NOS3, VEGFA expression	Angiogenic effects on diabetes	Wu et al. (2015)
			↓ Senescence		
**HCMECs**	1 μM	Hypoxia	↓ PAR-2, MAPK1/2, NF-κB expression	Anti-fibrotic and anti-oxidant effects	Imano et al. (2018)
**HCMECs**	1 μM	Hypoxia + sugen5416	↓ PAR-2, p-JNK, p-SMAD3, p-ERK-1/2, p-NF-kB expression	Anti-fibrotic effects	Imano et al. (2021)
**HUVECs**	920 nM	Hypoxia-reoxygenation (H/R)	No effect on *ICAM1 and VCAM1, THBD, and EPCR* expression	No protective impact on H/R conditions	Guillou et al. (2020)
**HUVECs**	229, 1150 nM	25-hydroxycholesterol (25 μΜ)	↓Endothelial permeability, *TF, ICAM1, VEGFA, IL-33, CCL2, TNF* expression	Stabilizes endothelial integrity, anti-inflammatory effects	Gorzelak-Pabis et al. (2021)
			↑ CDH5 expression		
**HUVECs**	1 μM	FXa (10 nM) + LPS	↓ PAR-2, NF-κB, IL-1b, TNF, IL-6, p-MAP3K7, p-P65 expression, apoptosis, migration, permeability	Anti-inflammatory effects on ALI	Shi et al. (2018)
			↑Viability		
**HPMEC**	50 nM	LPS (1 μg/mL) + SARS-CoV-2 Subunits S1 and S2 (10 nM)	↑ Mitochondrial membrane potential	Covid-19-induced mitochondrial shift prevention effects	Zekri-Nechar et al. (2022b)
			↓Cytochrome C oxidase activity, LDH activity, UCP2 expression		
**HBEC-5i + Human Astrocytes**	10 μM	NA	P-gp and BRCA-mediated BBB transportation	ABC transportation	Puech et al. (2018)

EA.hy926, Immortalized Human Umbilical Vein Endothelial Cell Line; HUVECs, Human Umbilical Vein Endothelial Cells; HaECs, Human Aortic Endothelial Cells; HCAECs, Human Coronary Artery Endothelial Cells; HDBECs, Human Dermal Blood Endothelial Cells; HCMECs, Human Cardiac Microvascular Endothelial Cells; EPCs, Endothelial Progenitor Cells; HPMEC, Human Pulmonary Microvascular Endothelial Cells; HBEC-5i, Human Brain Endothelial Cells-5 immortalized; FXa, Factor Xa; AGEs, Advanced Glycation End Products; LPS, Lipopolysaccharide; SARS-CoV-2, Severe Acute Respiratory Syndrome Coronavirus 2; CDKN2A, Cyclin-Dependent Kinase Inhibitor 2A; CDKN1C, Cyclin-Dependent Kinase Inhibitor 1C; EGR1, Early Growth Response 1; IGFBP-5, Insulin-Like Growth Factor-Binding Protein 5; IL-1β, Interleukin-1 beta; CCL2, C-C Motif Chemokine Ligand 2; ICAM1, Intercellular Adhesion Molecule-1; VCAM1, Vascular Cell Adhesion Molecule-1; MMP2, Matrix Metalloproteinase-2; EDN2, Endothelin-2; SELE, Selectin E; u-PA, urokinase Plasminogen Activator; MOK, Mitogen-Activated Protein Kinase Kinase; ROS, Reactive Oxygen Species; THP-1, Human Monocytic Cell Line; TFPI, Tissue Factor Pathway Inhibitor; NOx, Nitrogen Oxides; NOS3, Nitric Oxide Synthase 3; PRKN, Parkin RBR E3 Ubiquitin Protein Ligase; PINK1, PTEN-Induced Kinase 1; AKT1, Protein Kinase B; VEGFA, Vascular Endothelial Growth Factor A; PAR, Proteinase-Activated Receptor; p-MAPK1/2, Phosphorylated Mitogen-Activated Protein Kinase 1/2; NF-κB, Nuclear Factor Kappa B; JNK, c-Jun N-Terminal Kinase; SMAD3, Mothers Against Decapentaplegic Homolog 3; ERK-1/2, Extracellular Signal-Regulated Kinase 1/2; EPCR, Endothelial Protein C Receptor; TF, Tissue Factor; TNF, Tumor Necrosis Factor; CDH5, Cadherin 5; LDH, Lactate Dehydrogenase; UCP2, Uncoupling Protein 2; P-gp, P-Glycoprotein; BRCA, Breast Cancer Resistance Protein; BBB, Blood-Brain Barrier; ALI, Acute Lung Injury; COVID-19, Coronavirus Disease 2019; ABC, ATP-Binding Cassette.

FXa has been extensively used to investigate the molecular mechanisms underlying the effects of rivaroxaban in ECs. Lange et al. (2014) compared the anti-angiogenic action of inactive and active forms of FX using tubule formation assay in endothelial EA.hy926 cells. They showed that the anti-angiogenic action of FXa, but not FX, was reversed by rivaroxaban. These findings suggest that rivaroxaban can promote angiogenesis by inhibiting FXa activity in ECs (Lange et al., 2014).

Sanada et al. (2016) extensively studied the underlying mechanism of how ECs respond to chronic FXa-induced inflammation and cellular senescence, common features of tissues impacted by long-lasting inflammatory disorders. They treated HUVECs with FXa for 14 days and found that ECs senescence and growth retardation were triggered, whereas cell proliferation was suppressed. Furthermore, the mRNA expression of four genes, cyclin dependent kinase inhibitor 2A (*CDKN2A*), cyclin dependent kinase inhibitor 1C (*CDKN1C*), early growth response 1 (*EGR1*), insulin like growth factor binding protein 5 (*IGFBP-5*), as well as four inflammatory cytokines *IL-1β, IL-6, CCL2, ICAM1* were significantly enhanced. All of the above-mentioned FXa-induced actions were reversed by rivaroxaban. Additionally, they showed that ECs senescence is triggered by FXa through an IGFBP-5-dependent process. It appears that IGFBP-5 overexpression, which is not inhibited by rivaroxaban, leads to the upregulation of *IL-1β, IL-6*, and *ICAM1*. These results lead to the conclusion that rivaroxaban exhibits anti-inflammatory and angiogenic actions, that can reverse the effects of FXa, but not those of *IGFBP-5*, providing evidence that *IGFBP-5* is a downstream regulator of FXa-PAR signaling (Sanada et al., 2016).

The study by Seki et al. (2017) examined the role of rivaroxaban in FXa-induced inflammation in HUVECs. Rivaroxaban alone did not affect the expression of PARs or other pro-inflammatory genes. The addition of FXa to the medium resulted in upregulation of the expression of *PAR-1, -2,* and *-3* (but, notably, not −4), an effect that was reversed by rivaroxaban. Moreover, FXa stimulation led to mRNA overexpression of *ICAM1, CCL2*, and *IL-8*, all of which were inhibited by rivaroxaban. These results suggest that rivaroxaban can be beneficial in preventing FXa-induced progression of atherosclerosis by suppressing pro-inflammatory molecules (Seki et al., 2017).

The molecular mechanism behind the crosstalk of coagulation and inflammation and the potential beneficial effects of rivaroxaban were studied in cultured primary human aortic ECs (HaECs). FXa-induced mRNA upregulation of the inflammatory response genes MMP2, IL-1β, IL-6, IL-8, CCL2, ICAM1, and vascular cell adhesion molecule 1 (VCAM1) was abolished by rivaroxaban. In a monocyte-endothelial cell interaction assay, FXa induced monocyte adhesion to the endothelium, an effect that was also inhibited by rivaroxaban. These findings further support the idea that rivaroxaban possesses anti-inflammatory properties (Ding et al., 2021).

Another study on the effects of rivaroxaban in FXa-activated HUVECs was performed by Álvarez et al. (2018). Rivaroxaban alone boosted ECs viability, growth, and wound healing. It also led to differential expression of *MMP2* and urokinase-type plasminogen activator (*u-PA*) genes and reversed FXa-induced overexpression of the pro-inflammatory genes endothelin 2 (*EDN2*), selectin E (*SELE*), *VCAM1*, and *CCL5*. Furthermore, rivaroxaban dose-dependently inhibited FXa-induced platelet adhesion to HUVECs and stimulated u-PA activation in cell lysates and overexpression in the supernatants. All of these actions were reversed by a u-PA inhibitor. These findings suggest that the beneficial anti-inflammatory effects of rivaroxaban may be mediated by the activation of u-PA (Álvarez et al., 2018).

Benelhaj et al. (2019) used human coronary artery primary ECs (HCAEC) and human dermal blood-microvascular ECs (HDBEC) to study the effect of rivaroxaban on endothelial permeability. When treated with FXa, the ECs permeability in both models was reduced in the first 30 min and then increased at 60 min; rivaroxaban reversed both effects. Additional experiments revealed that these actions were mediated through PAR-1/-2 signaling pathways. These results indicate that rivaroxaban can maintain endothelial permeability at its physiological state (Benelhaj et al., 2019).

Hyperglycemia and diabetes are conditions that are closely related to microvascular inflammation and endothelial dysfunction. Several studies have been performed to investigate the effects of rivaroxaban in *in vitro* diabetic models. Ishibashi et al. (2014) assessed rivaroxaban’s effect in HUVECs exposed to advanced glycation end products (AGEs). Rivaroxaban reduced the production of citrated plasma-induced ROS in HUVECs in a dose-dependent manner. Additionally, AGEs exacerbated plasma-induced ROS production in HUVECs, which was diminished by treatment with rivaroxaban. Rivaroxaban reduced the upregulation of MOK protein kinase (*MOK*), *CCL2*, and *ICAM1* mRNA expression in HUVECs induced by citrated plasma and AGEs. Finally, 30 nM rivaroxaban minimized the effect of citrated plasma, which promoted THP-1 cell adhesion to HUVECs. These results reveal that rivaroxaban possesses anti-inflammatory and antioxidant properties on cultured ECs models of diabetes (Ishibashi et al., 2014).

The team of Yang et al. (2017) used a similar *in vitro* diabetic model of AGE-induced HUVECs to assess the occurrence of bleeding events of rivaroxaban co-administered with angiotensin II (Ang II). They found that this combination elevated tissue factor pathway inhibitor (*TFPI*) gene expression and activity, an effect that could not be produced by either treatment alone. Overall, their results suggest that *TFPI* is presumably a crucial mediator of Ang II enhancement of rivaroxaban anticoagulant actions via angiotensin II receptor type 2 (AT2R) and Mas signaling, implying an interaction between coagulation and the renin-angiotensin-aldosterone system (Yang et al., 2017).

Maeda et al. (2019) investigated the effects of rivaroxaban on a diabetic endothelial senescence model. When HUVECs were treated with high glucose, the cellular senescence-related proteins p53, p21, and CDKN2A were upregulated; rivaroxaban co-administration inhibited the overexpression of p53 and p16 (but not CDKN2A). When HUVECs were exposed to high glucose levels, telomerase activity, and telomere length were significantly decreased; rivaroxaban co-exposure mitigated these effects. Furthermore, rivaroxaban significantly reduced the protein expression of ICAM1, VCAM1, and p22^phox^, which were all upregulated by hyperglycemia. Rivaroxaban also diminished the ability of high glucose to increase ROS. In addition, it increased the concentration of products related to nitric oxide and nitric oxide synthase 3 (NOS3) protein expression caused by high glucose levels. Finally, rivaroxaban also mitigated hyperglycemia-induced PAR-1 protein levels. These findings suggest that rivaroxaban possesses anti-atherosclerotic and anti-senescence properties in HUVECs (Maeda et al., 2019).

Zekri-Nechar et al. (2022a) investigated the effects of the co-administration of rivaroxaban and aspirin in glucose-induced Human Coronary Artery Endothelial Cells (HCAECs) on mitochondrial mitophagy. Glucose-induced HCAECs had increased expression of FXa and tissue factor (TF), enhanced ROS production, and reduced mitochondrial membrane potential. Rivaroxaban reversed only FXa expression. Aspirin, on the other side, reversed all of these effects. Glucose also inhibited the protein expression of mitophagy proteins parkin RBR E3 ubiquitin-protein ligase (PRKN) and PTEN-induced kinase 1 (PINK1); an action that was reversed by aspirin but was unaffected by rivaroxaban alone. Interestingly, co-administration of rivaroxaban enhanced the effect of aspirin. These findings show that rivaroxaban and aspirin combined can potentially support the endothelium from hyperglycemic conditions due to mitochondrial mitophagy protection (Zekri-Nechar et al., 2022a).

Wu et al. (2015) by using *in vivo* and *in vitro* models of diabetes aimed to examine the impact of rivaroxaban in angiogenesis. They carried out scratch injury, tube formation, and senescence assays using human endothelial progenitor cells (EPCs) that had been preconditioned with a hyperglycemic medium. Cell viability assay showed that rivaroxaban alone enhanced EPCs proliferation compared to untreated control. Rivaroxaban also stimulated EPCs migration and tube formation, both of which were inhibited by high glucose conditions. Under the same conditions, rivaroxaban stimulated NOS3 phosphorylation, AKT serine/threonine kinase 1 (AKT1), and VEGFA protein expression in EPCs. These findings imply that rivaroxaban can support vascular function and has angiogenic properties at high glucose levels (Wu et al., 2015).

Hypoxia is a deficiency of oxygen supply in tissues and can occur in a range of CVDs and pulmonary diseases, such as ischemic heart disease, heart failure, obstructive sleep apnea, and pulmonary arterial hypertension. In both *in vivo* and *in vitro models*, hypoxia has been linked with fibrosis, inflammatory response, endothelial dysfunction, and other pathological conditions. Imano et al. (2018) have examined the potential pleiotropic effects of rivaroxaban in intermittent hypoxia, utilizing Human Cardiac Microvascular ECs (HCMECs). They showed that the mRNA and protein expression of *PAR-2*, mitogen-activated protein kinase 1/2 (*MAPK1/2*), and nuclear factor kappa-light-chain-enhancer (*NF-kB*) were upregulated under hypoxia compared to normoxia, and rivaroxaban, as well as PAR-2 antagonists, were able to restore their expression. These findings suggest that rivaroxaban can protect HCMECs from oxidative stress by blocking PAR-2-mediated activation of the ERK and NF-kB pathways (Imano et al., 2018).

Another study by this team assessed the impact of rivaroxaban on pulmonary arterial hypertension in the same hypoxia model. By treating HCMECs with sugen5416, a multi-kinase inhibitor of the VEGF receptor, under hypoxic settings, the investigators produced a model of rapid right ventricular remodeling. They found that PAR-2, phosphorylation of JNK, and SMAD family member 3 (SMAD3), as well as of mitogen-activated protein kinase 1/3 (MAPK1/3), and NF-kB were increased in this model, and the presence of rivaroxaban alleviated it. These results imply that rivaroxaban may be able to prevent fibrosis, ventricular hypertrophy, and RV remodeling by suppressing the MAPK and NF-kB pathways (Imano et al., 2021).

Guillou et al. (2020) carried out hypoxia-reoxygenation experiments in HUVECs in order to assess the potential effects of rivaroxaban in acute myocardial infarction. Rivaroxaban did not affect hypoxia-reoxygenation modulation of gene expression of *ICAM1, VCAM1, THBD*, and protein C receptor (*PROCR*). Thus, rivaroxaban appears to have no protective effect on ECs under hypoxia/reoxygenation conditions (Guillou et al., 2020).

A study addressing the effects of rivaroxaban on endothelial integrity and inflammation due to atherosclerosis was performed by Gorzelak-Pabis et al. (2021). Exposure of HUVECs to 25-OHC enhanced the mRNA expression of *TF, ICAM1, VEGFA, TNF, IL-33,* and *CCL2,* as well as endothelial permeability, and decreased CDH5 protein expression; all these effects were reversed by rivaroxaban. Thus, rivaroxaban can protect against atherosclerosis through its anti-inflammatory actions and protection of endothelial integrity (Gorzelak-Pabis et al., 2021).

In order to investigate how coagulation contributes to acute lung injury, Shi et al. (2018) treated HUVECs with lipopolysaccharide (LPS) -a microbial sepsis mediator- and FXa. Their experiments showed that rivaroxaban enhanced cell viability, inhibited apoptosis, and protected HUVECs from LPS-induced injury *in vitro*. In a wound healing assay, they demonstrated that LPS and FXa promoted HUVECs migration, whereas the addition of rivaroxaban considerably slowed this process. Additionally, LPS and FXa aggravated ECs permeability, which was reduced by rivaroxaban. In response to LPS and FXa treatments, HUVECs also secreted the cytokines IL-1b, TNF, and IL-6, while rivaroxaban suppressed this inflammatory response. Finally, they showed that rivaroxaban inactivates PAR-2 signaling and subsequent NF-B pathway activation, by deterring mitogen-activated protein kinase kinase 7 (MAP3K7) and p65 from being phosphorylated in LPS-induced HUVECs. These results suggest that rivaroxaban inhibits acute lung damage and LPS-induced inflammation through inactivation of the PAR-2/NF-B signaling pathway (Shi et al., 2018).

Zekri-Nechar et al. (2022b), studied the effects of severe acute respiratory syndrome coronavirus 2 (SARS-CoV-2) and FXa on mitochondrial metabolism in human pulmonary microvascular endothelial cells (HPMEC). LPS- and FXa-treated HPMECs were incubated with SARS-CoV-2 spike protein subunits S1 and S2, leading to a reduction in mitochondrial membrane potential, an effect that was partially reduced by rivaroxaban. Under the same conditions, the activities of cytochrome C oxidase and LDH, and the expression of uncoupling protein 2 (UCP2) were increased; the addition of rivaroxaban reduced both effects. These findings show that rivaroxaban lessens the SARS-CoV-2 spike S1 and S2 subunits-induced mitochondrial modification to anaerobic metabolism under pro-inflammatory settings via the participation of FXa inhibition (Zekri-Nechar et al., 2022b).

From a different perspective, Puech et al. (2018) established the HBEC-5i ECs as a model to investigate the mechanism of drug penetration through the human BBB. They found that rivaroxaban is transported by two ATP-binding cassette (ABC) transporters in their BBB model, namely, human breast cancer resistance protein (BCRP) and P-glycoprotein (P-GP). It appears that BCRP and P-GP transporters hold a significant role in how rivaroxaban is distributed in different tissues and are significant contributors to rivaroxaban pharmacokinetic variability (Puech et al., 2018).

### 4.3 Apixaban

Apixaban is a direct FXa inhibitor, approved by FDA in 2012 for stroke prevention in NV-AF, for VTE and PE treatment, and for VTE prevention after knee or hip replacement surgery (Frost et al., 2013). Apixaban appears to be superior in terms of safety and effectiveness, as it is the only DOAC to be associated with a lower risk of major bleeding or stroke compared to warfarin (Gupta et al., 2019). Additionally, it is also the oral anticoagulant of choice for patients with advanced chronic kidney disease, since it is the least dependent DOAC on renal metabolism (Xu et al., 2021). Apixaban binds to the active site of FXa, and its metabolism is influenced by CYP3A4 (Stacy et al., 2016). To date, only two studies have investigated the effects of apixaban on cultured human ECs, which are briefly presented in Table 3.

**TABLE 3:** Overview of the pleiotropic effects of apixaban in endothelial cells (ECs) *in vitro*.

Endothelial cell type	Apixaban dose	Stimulation factor	Cellular effects of apixaban	Biological/Pleiotropic effects	Reference
**HaECs**	10, 30, 100 nM	AF hemodynamics	↓ Fibrin deposition, thrombin generation	Clot resistance to fibrinolysis	Simmers et al. (2016)
			↑Fibrin density		
**HUVECs and HMEC-1**	130 nM	Uremic serum	↓VCAM1, ICAM1 expression, p- P38MAPK, p-P42/44	Anti-inflammatory effect	Torramade-Moix et al. (2021)
			↓ROS generation	Anti-oxidant effect	
			↑NOS3 expression	↓ Thrombogenicity	
			↓VWF expression, platelet adhesion		

HaECs, Human Aortic Endothelial Cells; HUVECs, Human Umbilical Vein Endothelial Cells; HMECs, Human Microvascular Endothelial Cells; AF, Atrial Fibrillation; VCAM1, Vascular Cell Adhesion Molecule-1; ICAM1, Intercellular Adhesion Molecule-1; P38MAPK, p38 Mitogen-Activated Protein Kinase; ROS, Reactive Oxygen Species; NOS3, Nitric Oxide Synthase 3; VWF, von Willebrand Factor.

Simmers et al. (2016) studied the effect of apixaban on fibrin deposition in a human ECs AF hemodynamic model. This model consists of a monolayer of HaECs cultured in a trans-well setup, exposed to AF hemodynamic flow, which allows the observation of images within a Z-stack at the surface of the trans-well until the top of the fibrin deposition. They found that apixaban lessens the fibrin deposition thickness while raising the fibrin density at the surface of ECs in this *in vitro* ECs AF hemodynamic model. These results indicate that apixaban can help mitigate the increased thrombotic risk associated with AF (Simmers et al., 2016).

Since apixaban is a safe choice for patients with end-stage kidney disease, Torramade-Moix et al. (2021) examined the protective role of apixaban in uremia-induced inflammation in an *in vitro* model of human dermal microvascular ECs (HMEC-1) and HUVECs. They exposed both ECs in uremic sera that caused endothelial dysfunction by increasing VCAM1, ICAM1, and ROS production, and reducing NOS3 and von Willebrand factor (VWF) expression, platelet adhesion, and phosphorylation of p38MAPK and p42/44. Apixaban abolished all these effects. These observations suggest that apixaban acts as an anti-inflammatory and antioxidant agent, can counteract uremic serum-induced endothelial cell dysregulation, and can be beneficial for patients with renal dysfunction (Torramade-Moix et al., 2021).

### 4.4 Edoxaban

Edoxaban, a direct Xa inhibitor, is the last DOAC approved by FDA in 2015 for stroke prevention in AF patients and VTE and PE treatment. Edoxaban acts by binding to the FXa active site and forms hydrogen bonds with Gly218 (Du et al., 2020). The effects of edoxaban on ECs are briefly presented in Table 4.

**TABLE 4:** Overview of the pleiotropic effects of edoxaban in endothelial cells (ECs) *in vitro*.

Endothelial cell type	Edoxaban dose	Stimulation factor	Cellular effects of edoxaban	Biological/Pleiotropic effects	Reference
**HUVECs**	1 nM ‐ 1 μM	-	↑ Viability	Proliferative action	Almengló et al. (2020)
	100–500 nM	FXa (9 nM)	↓ *ICAM1, VCAM1, SELE, PAI-1,* AREG, GRN, PLAU, SERPINE1 expression	Anti-inflammatory effects	
			↑ *PI3K*, CSF1, ENPP2, ERBB4 expression		
			↓ PBMCs and platelet adhesion, transmigration		
		—	↑ Fibrin formation	Hemostasis control	
			↑ u-PA activation		
		FXa (9 nM)	↑ Tube formation	Angiogenic effects	
			↓Migration	Anti-promigratory effects	

HUVECs, Human Umbilical Vein Endothelial Cells; FXa, Factor Xa; ICAM1, Intercellular Adhesion Molecule-1; VCAM1, Vascular Cell Adhesion Molecule-1; SELE, Selectin E; PAI-1, Plasminogen Activator Inhibitor-1; AREG, Amphiregulin; GRN, Granulin Precursor; PLAU, Plasminogen Activator Urokinase; SERPINE1, Serpin Family E Member 1; PI3K, Phosphoinositide 3-Kinase; CSF1, Colony Stimulating Factor 1; ENPP2, Ectonucleotide Pyrophosphatase/Phosphodiesterase 2; ERBB4, Erb-B2 Receptor Tyrosine Kinase 4; PBMCs, Peripheral Blood Mononuclear Cells; u-PA, urokinase Plasminogen Activator.

There is a dearth of publications investigating the effects of edoxaban on ECs *in vitro*. In the only available study, the actions of edoxaban on HUVECs proliferation, migration, angiogenesis, inflammation, and coagulation were assessed. Edoxaban alone promoted cell viability and growth. Fibrin formation was not affected by edoxaban in a cell-free assay but was considerably enhanced when HUVECs were present. In FXa-treated ECs, edoxaban showed anti-inflammatory properties by blocking peripheral blood mononuclear cell (PBMC) and platelet adhesion to HUVECs, while effectively reversing the transmigration of PBMCs through HUVECs monolayers caused by FXa. Edoxaban also protected the endothelium by attenuating FXa-induced migration in a wound healing assay and by partially neutralizing the anti-angiogenic effects of FXa. In protein expression analysis, edoxaban compared to control led to VEGFA, EPCAM, MMP2, amphiregulin (AREG) downregulation and dickkopf-related protein 1 (DKK1), delta-like protein 1 (DLL1), mammalian STE20-like protein kinase 1 (MST1), erb-b2 receptor tyrosine kinase 4 (ERBB4), and interleukin-2 receptor subunit alpha (IL2RA) upregulation. The addition of edoxaban to FXa-treated ECs led to the underexpression of several proteins, including VCAM1, granulin precursor (GRN), plasminogen activator urokinase (PLAU), and serpin family E member 1 (SERPINE1), and overexpression of several other proteins, including colony stimulating factor 1 (CSF1), ectonucleotide pyrophosphatase/phosphodiesterase 2 (ENPP2), and ERBB4. In cells treated with FXa, edoxaban downregulated the gene expression of *ICAM1, VCAM1, SELE,* and *PAI-1* and upregulated *PI3K*. Both protein and expression analyses revealed molecules associated with the PI3K/AKT signaling pathway, interleukin signaling, or the immune system. These findings suggest that edoxaban exerts anti-inflammatory effects on the endothelium and enhances ECs growth while also controlling hemostasis, actions mediated mainly via the PAR-1-2/PI3K/NF-kB pathway (Almengló et al., 2020).

### 4.5 Comparative studies of DOACs

Several studies have compared DOACs with one another in ECs *in vitro* models to evaluate their potential differences and similarities. Considering that FXa inhibitors share the same mechanism of action, the majority of studies have compared dabigatran, which acts through the inhibition of thrombin, with the other three agents that inhibit FXa. The pleiotropic effects of DOACs in comparative studies on *in vitro* ECs are presented in Table 5.

**TABLE 5:** Overview of the pleiotropic effects of DOACs in endothelial cells (ECs) *in vitro*.

Endothelial cell type	DOACs dose	Stimulation factor	Physiological effects	Biological/Pleiotropic effects	Reference
**OECs and HUVECs**	Dabigatran (0,1–20 μM), Rivaroxaban (0,1–20 μM)	Thrombin (80 nM) or FXa (50 nM)	↓ ICAM1 membrane expression	Anti-inflammatory effects	Papadaki et al. (2020)
			↓ CCL2 secretion		
**HUVECs**	Rivaroxaban (0,1–3 μM)	Thrombin (10 nM)	*↓ SELE, ICAM1, VCAM1, IL-8, CCL2, CXCL1, CXCL2, TF*	Anti-inflammatory effects	Ellinghaus et al. (2016)
	Dabigatran (0,1–3 μM)				
	Dabigatran (10–30 nM)		↑*CXCL1, CXCL2, IL-8, SELE, CCL2, TF*	Pro-inflammatory effect	
**HUVECs**	Dabigatran (160, 800 nM), Rivaroxaban (230, 1150 nM)	25-hydroxycholesterol (25 μM)	↑ *TGFB1, IL-37*	Anti-inflammatory effects	Gorzelak-Pabiś et al. (2022b)
			↓ *IL-18, IL-23,* and *IL-35*		
			Repair DNA single-strand breaks	Protect from DNA damage	Woźniak et al. (2020)
			↓ ROS generation	Anti-oxidant effects	
**HBEC-5i + Human Astrocytes**	Dabigatran (500 nM), Rivaroxaban (500 nM), Apixaban (500 nM)	Thrombin (100 nM)	↓ Permeability, PAR-1 cleavage	Stabilizes BBB integrity and anti-hemorrhagic effects	Puech et al. (2019)
			↑TJP1, CDH5 expression		
**EA.hy926**	Dabigatran (200 nM)	Anti-TM, Anti-EPCR, Anti-APC	Non-APC signaling activation	Non- APC mediating bleeding	von Drygalski et al. (2020)
	Rivaroxaban (200 nM), Apixaban (200 nM), Edoxaban (200 nM)		APC signaling activation	APC mediating bleeding	

OECs, Outgrowth Endothelial Cells; HUVECs, Human Umbilical Vein Endothelial Cells; HBEC-5i, Human Brain Endothelial Cells-5 immortalized; EA.hy926, Immortalized Human Umbilical Vein Endothelial Cell Line; TM, Thrombomodulin; EPCR, Endothelial Protein C Receptor; APC, Activated Protein C; ICAM1, Intercellular Adhesion Molecule-1; CCL2, C-C Motif Chemokine Ligand 2; SELE, Selectin E; VCAM, Vascular Cell Adhesion Molecule; IL-8, Interleukin 8; CXCL2, C-X-C Motif Chemokine 2; TF, Tissue Factor; TGFB1, Transforming Growth Factor Beta 1; ROS, Reactive Oxygen Species; PAR, Proteinase-Activated Receptor; TJP1, Tight Junction Protein 1; CDH5, Cadherin 5.

Most comparative studies of DOACs have been performed with dabigatran and rivaroxaban. Papadaki et al. (2020) studied the impact of these two DOACs on ECs activation by thrombin and FXa. In human late-outgrowth ECs (OECs) and HUVECs, both FXa and thrombin promoted ICAM1 expression and CCL2 secretion. Dabigatran and rivaroxaban ameliorated thrombin and FXa-induced effects respectively, in a dose-dependent manner. Additionally, rivaroxaban treatment promoted thrombin-induced ICAM1 expression and CCL2 secretion. Altogether, these observations reveal that both dabigatran and rivaroxaban exert similar anti-inflammatory properties (Papadaki et al., 2020).

Another study assessed the impact of these two DOACs on endothelium-induced inflammation. They induced HUVECs with thrombin with and without a PAR-1 antagonist (vorapaxar) and performed microarray experiments to identify the top 20 differentially expressed genes in response to this stimulus. The most strongly upregulated thrombin-induced genes were *SELE, VCAM1, ICAM1, CCL2, IL-8, CXCL1,* and *CXCL2.* When plasma from patients who had received rivaroxaban and dabigatran was added, the expression of these inflammatory genes was attenuated in a dosage-dependent manner. However, it should be noted that dabigatran at very low doses stimulated the expression of *CXCL1, CXCL2, IL-8, ELAM-1, CCL2,* and *TF*. These findings suggest that while both DOACs are equally effective in alleviating the pro-inflammatory responses caused by PAR-1 activation, some differences in their actions may exist (Ellinghaus et al., 2016).

Gorzelak-Pabiś et al. (2022b) also evaluated the effects of the same DOACs in a HUVECs inflammation model. Stimulation of HUVECs with 25-OHC caused downregulation of transforming growth factor beta 1 (*TGFB1*) and IL-37, and upregulation of *IL-18, IL-23,* and *IL-35,* which were counterbalanced when ECs were pre-incubated with rivaroxaban or dabigatran. Thus, both drugs exert anti-inflammatory properties (Gorzelak-Pabiś et al., 2022b). The same team also looked into the effects of dabigatran and rivaroxaban on DNA oxidative damage in HUVECs induced by 25-OHC. They found increased DNA oxidative damage using the comet assay, while they also measured enhanced ROS generation using flow cytometry. Both actions were reduced by rivaroxaban and dabigatran, with the latter exhibiting a stronger effect at higher doses than rivaroxaban. These results indicate that both drugs indirectly protect DNA by suppressing the formation of ROS, with dabigatran having a stronger antioxidant effect than rivaroxaban (Woźniak et al., 2020).

Intracerebral hemorrhage is one of the major and life-threatening complications of DOACs. A study investigated the impact of dabigatran, rivaroxaban, and apixaban in a BBB model of HBEC-5i ECs impaired by thrombin by measuring permeability, junction protein expression, such as tight junction protein 1 (TJP1) and CDH5, and PAR-1 cleavage. All three DOACs blocked the modification of endothelial permeability by sparing the thrombin-inhibited reduction of TJP1 and CDH5 expression and hindered PAR-1 cleavage. Overall, it appears that DOACs protect BBB from damage brought on by thrombin-induced PAR-1 activation (Puech et al., 2019).

From a different angle, von Drygalski et al. (2020) examined a novel agent that counteracts the hemorrhagic effects of DOACs. When all FDA-approved DOACs (rivaroxaban, apixaban, edoxaban, and dabigatran) were added to EA.hy926ECs as part of an endothelial thrombin generation assay, they discovered that thrombin generation was dramatically reduced (7-to-8-fold change) compared to the absence of ECs. Next, they added antibodies against THBD, endothelial protein C receptor (EPCR), and activated protein C (APC), which increased the production of thrombin in ECs treated with rivaroxaban, edoxaban, and apixaban, but not dabigatran. These findings point out the involvement of the APC coagulation cascade only in FXa inhibitor-associated bleedings (von Drygalski et al., 2020).

## 5 Conclusion and perspectives

It is well-established that endothelial dysfunction is a principal cause of CVDs. In cardiovascular research, cultured ECs have been used extensively in the past and continue to provide a useful and reliable *in vitro* model for the study of the pathophysiological mechanisms of CVDs. Additionally, cultured ECs can be used to aid in the development of novel drugs targeting the vasculature and in the study of the pleiotropic effects of drugs already on the market. DOACs are highly effective anticoagulants that offer a range of untapped potentials in modulating vascular functions (Rogula et al., 2022). Thus, assessing the effects of DOACs in cultures of ECs can provide valuable knowledge on their effects beyond anticoagulation *in vitro*, generating hypotheses that will eventually lead to the identification of the full spectrum of their protective and/or damaging behavior in the millions of patients receiving them.

**FIGURE 2:**
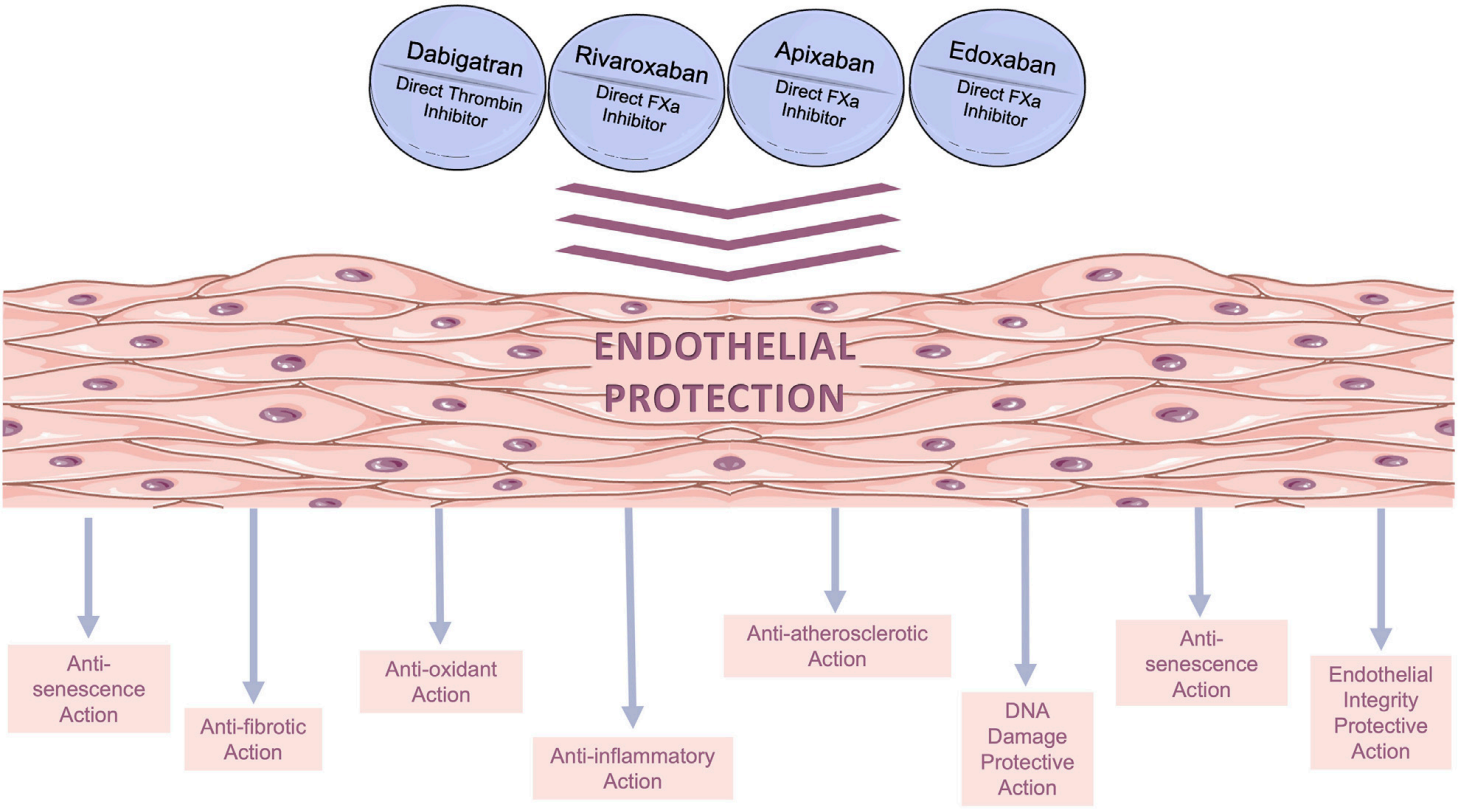
Beneficial pleiotropic effects of direct oral anticoagulants (DOACs) on *in vitro* human endothelial models. The main pleiotropic effects of DOACs are anti-oxidant, anti-inflammatory, anti-fibrotic, and anti-senescence effects, as well as decreased permeability.

DOACs have been used in clinical practice for more than a decade as a standard *per os* anticoagulation therapy. Following the examples of statins, metformin, and several other widely used cardiovascular drugs, a search for additional (pleiotropic) effects is currently underway in several preclinical and clinical studies. As it collectively derives from the studies reviewed herein, DOACs have several pleiotropic actions in ECs; it appears that apart from anticoagulation, they exert several additional beneficial actions on the endothelium (depicted in Figure 2), including anti-oxidant (Torramade-Moix et al., 2021), anti-inflammatory (Álvarez et al., 2018), atheroprotective (Gorzelak-Pabiś et al., 2022b), anti-senescence (Sanada et al., 2016), and anti-fibrotic effects (Imano et al., 2021). Moreover, they preserve endothelial integrity (Benelhaj et al., 2019; Gorzelak-Pabiś et al., 2022a; Zolotoff et al., 2022) and reduce thrombogenicity (Torramade-Moix et al., 2021). The non-anticoagulant effects of DOACs on ECs have also been evaluated *in vivo.* Specifically, studies in rodents have suggested that DOACs exert pleiotropic effects on ECs through PAR mediation confirming their existence (Lee et al., 2012; Pingel et al., 2014; Villari et al., 2017; Rahadian et al., 2020; Ito et al., 2021). Furthermore, ongoing investigations in clinical settings (Di Santo et al., 2023) have shed additional light on the pleiotropic effects of DOACs, and evidence has already emerged from clinical studies, indicating that DOACs improve cardiovascular health beyond their primary anticoagulant function (Pistrosch et al., 2021; Lin et al., 2023).

Efforts to develop safer and more effective DOACs are currently underway. The most promising class of compounds is FXIa inhibitors, which target the coagulation cascade upstream of the currently existing DOACs. Some of these compounds, such as asundexian and milvexian, are currently in phase 3 clinical trials, and there is evidence that they can inhibit thrombosis while preserving hemostasis (Schumacher et al., 2010; Muscente and De Caterina, 2023; Wichaiyo et al., 2023). Emerging preclinical evidence suggests that FXIa inhibitors may exert pleiotropic effects, such as anti-inflammatory and anti-atherogenic (Ngo et al., 2021). Pointing to this direction, a recent study has demonstrated that in cultured vascular smooth muscle cells, FXIa induces cellular responses through the activation of PARs (Liu et al., 2019). However, to the best of our knowledge, there are no published studies with FXIa inhibitors on *in vitro* ECs. Further research is needed to explore the potential effects of these agents on ECs and better understand their non-anticoagulant actions.

As previously mentioned, thrombin is a serine protease generated by the proteolytic activation of the zymogen prothrombin with the participation of FXa (Krishnaswamy, 2013). Thrombin mediates cellular responses through proteolytic activation of PAR signaling by cleaving the amino-terminal extracellular domain (exodomain) and uncovering new N-terminal peptides that function as tethered ligands for G protein-dependent receptors, thereby promoting signal transduction (Coughlin, 2000; Heuberger and Schuepbach, 2019). The pleiotropic actions of DOACs, such as anti-inflammatory, anti-oxidant, anti-fibrotic, and BBB integrity protection, are mediated mainly through the PAR signaling pathway. PARs trigger the transduction of several intracellular signals, through activating PI3K/Akt and NF-kB signaling pathways, and by extension leading to various cellular responses, including inflammation, endothelial integrity, and migration (Ishibashi et al., 2014; Sanada et al., 2016; Shi et al., 2018; Imano et al., 2021).

PAR signaling inhibition has been proven through the use of PAR inhibitors such as vorapaxar, siRNA, or antagonist peptides (Ellinghaus et al., 2016; Benelhaj et al., 2019; Maeda et al., 2019; Puech et al., 2019; Papadaki et al., 2020; Vittal Rao et al., 2021). Additionally, some studies have demonstrated the involvement of PARs through experiments evaluating PARs activation (Zolotoff et al., 2022) or expression (Imano et al., 2021). Nevertheless, there are some indications that DOACs may act beyond PAR signaling. Several studies have evaluated the actions of DOACs in the absence of stimulators and showed that ECs can be stimulated by DOACs and exert non-anticoagulant effects on endothelium off-target (Wu et al., 2015; Álvarez et al., 2018; Almengló et al., 2020). Several hypotheses can be suggested to explain these phenomena. They may act either through the activation of receptors and subsequent signaling transduction by DOACs, or by the binding of DOACs to off-target factors on the endothelium. Alternatively, they could be a result of the suppression of the constitutive expression of thrombin in endothelial cells through inhibition of the coagulation cascade. The underlying mechanisms and molecular pathways should be further investigated to determine the potential contribution of PAR-independent pathways.

Compared to VKAs, DOACs offer an advantage by inhibiting both thrombin and PAR signaling activation. On the other hand, VKAs can attenuate coagulation, but also lead to the production of coagulation factors known as proteins induced by vitamin K antagonism or absence (PIVKA). PIVKA are inactive as coagulation factors because they lack the Gla domain but retain their proteolytic activity, which can modulate PAR signaling (Spronk et al., 2014). The dual action of DOACs, which inhibits both the coagulation process and PAR-mediated signaling, provides a more comprehensive and efficient approach to anticoagulation, contributing to the beneficial therapeutic profile of the cardiovascular system compared to VKAs.

Four DOACs are currently in use worldwide. Clinicians are striving to personalize their clinical use by identifying factors that can distinguish the four compounds and provide information for optimal selection for each patient. Some pharmacokinetic differences are known and considered, but there is a lack of biomarkers for precision medication. Although most of the effects of all DOACs are similar, some indications of heterogeneity have emerged in the present detailed analysis of their effects on ECs *in vitro*, which we discuss below.

Extensive research has thoroughly assessed the clinical concentrations of DOACs for anticoagulation in AF patients, showing a wide therapeutic index. Daily administration of dabigatran results in peak plasma levels within the range of 62–447 ng/mL (approximately 100–712 nM) (van Ryn et al., 2010; van Ryn et al., 2013). Similarly, for rivaroxaban, patients exhibit peak serum levels ranging from 184 to 343 ng/mL (approximately 423–788 nM) (Mueck et al., 2014). In the case of apixaban, patients' serum levels vary between 14 and 716 ng/mL (approximately 30–1,558 nM) (Frost et al., 2013), whereas edoxaban yields peak serum levels ranging between 60 and 250 ng/mL (approximately 109–455 nM) (Weitz et al., 2010). The majority of studies in cultured endothelial cells presented in this review used concentrations of DOACs within the range of the achieved concentrations in patients’ serum levels in anticoagulation (100–500 nM) (Tables 1–5). However, there are studies evaluating lower levels to assess the onset of pleiotropic actions (0.1 nM), as well as studies investigating very high levels of DOACs to evaluate their potential toxicity (20 μM). DOACs can manifest non-anticoagulant actions at undertherapeutic levels (10, 30 nM) (Ishibashi et al., 2014; Vianello et al., 2016). Simultaneously, at hypertherapeutic concentrations (5–20 μM), they do not harm the endothelium and retain their beneficial actions (Wu et al., 2015; Sanada et al., 2016; Papadaki et al., 2020).

Thrombin has a dual role in ECs, and its low concentrations have beneficial effects on endothelial function, controlling the equilibrium of hemostasis-hemorrhage (García et al., 2015). Complete inhibition of thrombin on ECs with dabigatran compromises the endothelium (Smeda et al., 2022). Instead, FXa inhibitors function upstream in the coagulation cascade and may not block the low constitutive expression of thrombin, which is pivotal for ECs function. Furthermore, dabigatran appears to possess biphasic actions, promoting proinflammatory stimuli at very low concentrations, whereas FXa inhibitors do not have this function (Ellinghaus et al., 2016). Besides, very high doses of thrombin-bound dabigatran stabilized PAR-1 membrane expression and modulated its function (Chen et al., 2015). Concurrently, the thrombin-induced inflammatory response was enhanced by an FXa inhibitor (Papadaki et al., 2020), whereas FXa inhibitors do not fully suppress thrombin generation, enabling the activation of APC (von Drygalski et al., 2020). Previous studies have shown that direct thrombin inhibitors, such as dabigatran and melagatran, but not FXa inhibitors, enhance thrombin generation and hypercoagulability via thrombin-induced negative-feedback system through inhibition of the protein C system (Furugohri et al., 2011; Perzborn et al., 2014; Furugohri and Morishima, 2015). Both negative effects of dabigatran on ECs suggest that maintaining a delicate balance of thrombin activity is crucial for endothelial function. DOACs may have distinct effects on thrombin expression, endothelial function, and coagulation regulation, necessitating further investigation.

Furthermore, the angiogenic effects of DOACs on ECs are inconsistent. Three distinct studies using FXa inhibitors have documented increased tube formation, leading to improved angiogenesis (Lange et al., 2014; Wu et al., 2015; Almengló et al., 2020). In contrast, dabigatran, a direct thrombin inhibitor, was found to cause decreased tube formation (Vianello et al., 2016). This interesting observation deserves further study.

Some differences between DOACs in ECs have also been observed in relation to their effect on migration. The study by Shi et al. (2018) reported that rivaroxaban reduced FXA- and LPS-induced migration in HUVECs (Shi et al., 2018). Similarly, edoxaban neutralized FXa-induced migration of HUVECs (Almengló et al., 2020). In contrast, in hyperglycemia-suppressed EPCs, migration was slowed, and the addition of rivaroxaban enhanced migration (Wu et al., 2015). Also, in non-induced HUVECs, Álvarez et al. reported enhanced migration after rivaroxaban treatment (Álvarez et al., 2018). Although there are conflicting findings on the wound-healing abilities of FXa inhibitors, it seems that DOACs can counteract the effects of stimulants.

DOAC-related enhancement of cell growth is debatable. Studies are showing that DOACs promote cell growth and proliferation (Wu et al., 2015; Sanada et al., 2016; Shi et al., 2018; Álvarez et al., 2018; Almengló et al., 2020), while in other studies no such effect was found (Woźniak et al., 2020; Gorzelak-Pabis et al., 2021; Gorzelak-Pabiś et al., 2022a). These contradictory effects may uncover real differences or be the result of different experimental settings. Further investigation is necessary to answer this question.

Another contentious finding is the THBD expression after DOACs treatment. Noguchi et al. reported that dabigatran enhanced THBD protein expression on ischemia/reperfusion injury in SECs (Noguchi et al., 2021). Under the same conditions, on HUVECs, Guillou et al. found no significant effects of rivaroxaban on *THBD* and other genes (*ICAM1, VCAM1, EPCR*) (Guillou et al., 2020). This difference may be related to the different cell types and/or DOAC used and requires further evaluation.

Remarkably, dabigatran and rivaroxaban have been used so far in the majority of investigations conducted to study the effects of DOACs on ECs, whereas there is a dearth of studies regarding apixaban and edoxaban. Especially for apixaban, which has been introduced in the clinic at about the same time as rivaroxaban, this is difficult to comprehend. It would be an oversimplification to argue that since all three FXa inhibitors have identical targets and presumably identical mechanisms of action, no comparative studies are necessary. We suggest that more research should be conducted to determine how also apixaban and edoxaban affect ECs.

Likewise, further research is warranted to gain adequate knowledge of the mechanisms and the long-standing effects of DOACs on the vasculature, facilitating the translation of these findings into clinical relevance, and helping identify non-responders and individuals at risk of DOAC-related adverse effects (Palmirotta, 2022). Additionally, a more comprehensive understanding of the precise downstream cell signaling mechanisms can be achieved by employing pharmaco-omics approaches that go beyond transcriptomic and phenotypic changes (Ragia and Manolopoulos, 2022).

In conclusion, ECs offer valuable tools for studying the effects of DOACs beyond their anticoagulant properties. DOACs exhibit pleiotropic actions on ECs, such as anti-inflammatory, anti-atherosclerotic, and anti-fibrotic effects, as well as preservation of endothelial integrity. Further research is needed to fully understand the pleiotropic effects of DOACs on ECs, their underlying mechanisms, as well as potential differences between the various DOACs. Such studies can pave the way for identifying biomarkers helping to personalize pharmacotherapy with this very valuable class of drugs.

## References

[B1] Almengló C. Mosquera-Garrote N. González-Peteiro M. González-Juanatey J. R. Álvarez E. (2020). Edoxaban's contribution to key endothelial cell functions. Biochem. Pharmacol. 178, 114063. 10.1016/j.bcp.2020.114063 32492447

[B2] Álvarez E. Paradela-Dobarro B. Raposeiras-Roubín S. González-Juanatey J. R. (2018). Protective, repairing and fibrinolytic effects of rivaroxaban on vascular endothelium. Br. J. Clin. Pharmacol. 84 (2), 280–291. 10.1111/bcp.13440 28940408 PMC5777430

[B3] Borensztajn K. Peppelenbosch M. P. Spek C. A. (2009). Coagulation factor Xa signaling: the link between coagulation and inflammatory bowel disease? Trends Pharmacol. Sci. 30 (1), 8–16. 10.1016/j.tips.2008.10.007 19058861

[B4] Benelhaj N. E. Maraveyas A. Featherby S. Collier M. E. W. Johnson M. J. Ettelaie C. (2019). Alteration in endothelial permeability occurs in response to the activation of PAR2 by factor Xa but not directly by the TF-factor VIIa complex. Thromb. Res. 175, 13–20. 10.1016/j.thromres.2019.01.009 30677622

[B5] Chen B. Soto A. G. Coronel L. J. Goss A. van Ryn J. Trejo J. (2015). Characterization of thrombin-bound dabigatran effects on protease-activated receptor-1 expression and signaling *in vitro* . Mol. Pharmacol. 88 (1), 95–105. 10.1124/mol.114.096446 25934730 PMC4468637

[B6] Choi H. J. Kim N. E. Kim J. An S. Yang S. H. Ha J. (2018). Dabigatran reduces endothelial permeability through inhibition of thrombin-induced cytoskeleton reorganization. Thromb. Res. 167, 165–171. 10.1016/j.thromres.2018.04.019 29735342

[B7] Coughlin S. R. (2000). Thrombin signalling and protease-activated receptors. Nature 407 (6801), 258–264. 10.1038/35025229 11001069

[B8] Danckwardt S. Hentze M. W. Kulozik A. E. (2013). Pathologies at the nexus of blood coagulation and inflammation: thrombin in hemostasis, cancer, and beyond. J. Mol. Med. Berl. 91 (11), 1257–1271. 10.1007/s00109-013-1074-5 23955016 PMC3825489

[B9] Di Santo P. Abdel-Razek O. Jung R. Parlow S. Poulin A. Bernick J. (2023). Rationale and design of the rivaroxaban post-transradial access for the prevention of radial artery occlusion trial (CAPITAL-RAPTOR). BMJ Open 13 (5), e070720. 10.1136/bmjopen-2022-070720 PMC10186464 37173116

[B10] Ding Y. Li X. Zhou M. Cai L. Tang H. Xie T. (2021). Factor Xa inhibitor rivaroxaban suppresses experimental abdominal aortic aneurysm progression via attenuating aortic inflammation. Vasc. Pharmacol. 136, 106818. 10.1016/j.vph.2020.106818 33227452

[B11] Du Q. Qian Y. Yao X. Xue W. (2020). Elucidating the tight-binding mechanism of two oral anticoagulants to factor Xa by using induced-fit docking and molecular dynamics simulation. J. Biomol. Struct. Dyn. 38 (2), 625–633. 10.1080/07391102.2019.1583605 30806177

[B12] Ellinghaus P. Perzborn E. Hauenschild P. Gerdes C. Heitmeier S. Visser M. (2016). Expression of pro-inflammatory genes in human endothelial cells: Comparison of rivaroxaban and dabigatran. Thromb. Res. 142, 44–51. 10.1016/j.thromres.2016.04.008 27131284

[B13] Esmon C. T. (2014). Targeting factor Xa and thrombin: impact on coagulation and beyond. Thromb. Haemost. 111 (4), 625–633. 10.1160/th13-09-0730 24336942

[B14] Frost C. Nepal S. Wang J. Schuster A. Byon W. Boyd R. A. (2013). Safety, pharmacokinetics and pharmacodynamics of multiple oral doses of apixaban, a factor Xa inhibitor, in healthy subjects. Br. J. Clin. Pharmacol. 76 (5), 776–786. 10.1111/bcp.12106 23451769 PMC3853536

[B15] Furugohri T. Morishima Y. (2015). Paradoxical enhancement of the intrinsic pathway-induced thrombin generation in human plasma by melagatran, a direct thrombin inhibitor, but not edoxaban, a direct factor Xa inhibitor, or heparin. Thromb. Res. 136 (3), 658–662. 10.1016/j.thromres.2015.06.034 26188924

[B16] Furugohri T. Sugiyama N. Morishima Y. Shibano T. (2011). Antithrombin-independent thrombin inhibitors, but not direct factor Xa inhibitors, enhance thrombin generation in plasma through inhibition of thrombin-thrombomodulin-protein C system. Thromb. Haemost. 106 (6), 1076–1083. 10.1160/th11-06-0382 22012070

[B17] García P. S. Ciavatta V. T. Fidler J. A. Woodbury A. Levy J. H. Tyor W. R. (2015). Concentration-dependent dual role of thrombin in protection of cultured rat cortical neurons. Neurochem. Res. 40 (11), 2220–2229. 10.1007/s11064-015-1711-1 26342829 PMC4644093

[B18] Gorzelak-Pabiś P. Broncel M. Pawlos A. Wojdan K. Gajewski A. Chałubiński M. (2022a). Dabigatran: its protective effect against endothelial cell damage by oxysterol. Biomed. Pharmacother. 147, 112679. 10.1016/j.biopha.2022.112679 35121342

[B19] Gorzelak-Pabis P. Broncel M. Wojdan K. Gajewski A. Chalubinski M. Gawrysiak M. (2021). Rivaroxaban protects from the oxysterol-induced damage and inflammatory activation of the vascular endothelium. Tissue Barriers 9 (4), 1956284. 10.1080/21688370.2021.1956284 34323663 PMC8794498

[B20] Gorzelak-Pabiś P. Pawlos A. Broncel M. Wojdan K. Woźniak E. (2022b). Expression of anti and pro-inflammatory genes in human endothelial cells activated by 25-hydroxycholesterol: A comparison of rivaroxaban and dabigatran. Clin. Exp. Pharmacol. Physiol. 49 (8), 805–812. 10.1111/1440-1681.13668 35577580

[B21] Graf L. Tsakiris D. A. (2012). Anticoagulant treatment: the end of the old agents? Swiss Med. Wkly. 142, w13684. 10.4414/smw.2012.13684 23037610

[B22] Granger C. B. Alexander J. H. McMurray J. J. Lopes R. D. Hylek E. M. Hanna M. (2011). Apixaban versus warfarin in patients with atrial fibrillation. N. Engl. J. Med. 365 (11), 981–992. 10.1056/NEJMoa1107039 21870978

[B23] Grimsey N. J. Trejo J. (2016). Integration of endothelial protease-activated receptor-1 inflammatory signaling by ubiquitin. Curr. Opin. Hematol. 23 (3), 274–279. 10.1097/moh.0000000000000232 26845544 PMC4978167

[B24] Guillou S. Beaumont J. Tamareille S. Giraud S. Mirebeau-Prunier D. Prunier F. (2020). Direct rivaroxaban-induced factor XA inhibition proves to be cardioprotective in rats. Shock 53 (6), 730–736. 10.1097/SHK.0000000000001412 31348147

[B25] Gupta K. Trocio J. Keshishian A. Zhang Q. Dina O. Mardekian J. (2019). Effectiveness and safety of direct oral anticoagulants compared to warfarin in treatment naïve non-valvular atrial fibrillation patients in the US Department of defense population. BMC Cardiovasc Disord. 19 (1), 142. 10.1186/s12872-019-1116-1 31195999 PMC6567643

[B26] Hart R. G. Diener H. C. Yang S. Connolly S. J. Wallentin L. Reilly P. A. (2012). Intracranial hemorrhage in atrial fibrillation patients during anticoagulation with warfarin or dabigatran: the RE-LY trial. Stroke 43 (6), 1511–1517. 10.1161/strokeaha.112.650614 22492518

[B27] Heuberger D. M. Schuepbach R. A. (2019). Protease-activated receptors (PARs): mechanisms of action and potential therapeutic modulators in PAR-driven inflammatory diseases. Thrombosis J. 17 (1), 4. 10.1186/s12959-019-0194-8 PMC6440139 30976204

[B28] Imano H. Kato R. Nomura A. Tamura M. Yamaguchi Y. Ijiri Y. (2021). Rivaroxaban attenuates right ventricular remodeling in rats with pulmonary arterial hypertension. Biol. Pharm. Bull. 44 (5), 669–677. 10.1248/bpb.b20-01011 33612567

[B29] Imano H. Kato R. Tanikawa S. Yoshimura F. Nomura A. Ijiri Y. (2018). Factor Xa inhibition by rivaroxaban attenuates cardiac remodeling due to intermittent hypoxia. J. Pharmacol. Sci. 137 (3), 274–282. 10.1016/j.jphs.2018.07.002 30055890

[B30] Ishibashi Y. Matsui T. Ueda S. Fukami K. Yamagishi S. (2014). Advanced glycation end products potentiate citrated plasma-evoked oxidative and inflammatory reactions in endothelial cells by up-regulating protease-activated receptor-1 expression. Cardiovasc Diabetol. 13, 60. 10.1186/1475-2840-13-60 24624928 PMC3995632

[B31] Ito Y. Maejima Y. Nakagama S. Shiheido-Watanabe Y. Tamura N. Sasano T. (2021). Rivaroxaban, a direct oral factor Xa inhibitor, attenuates atherosclerosis by alleviating factor xa-PAR2-mediated autophagy suppression. JACC Basic Transl. Sci. 6 (12), 964–980. 10.1016/j.jacbts.2021.09.010 35024502 PMC8733676

[B32] Krishnaswamy S. (2013). The transition of prothrombin to thrombin. J. Thromb. Haemost. 11 (1), 265–276. 10.1111/jth.12217 23809130 PMC3713535

[B33] Kuehn B. M. (2021). First oral blood thinner is approved for children. JAMA 326 (7), 593. 10.1001/jama.2021.13259 34402847

[B34] Lange S. Gonzalez I. Pinto M. P. Arce M. Valenzuela R. Aranda E. (2014). Independent anti-angiogenic capacities of coagulation factors X and Xa. J. Cell Physiol. 229 (11), 1673–1680. 10.1002/jcp.24612 24615682

[B35] Lee I. O. Kratz M. T. Schirmer S. H. Baumhäkel M. Böhm M. (2012). The effects of direct thrombin inhibition with dabigatran on plaque formation and endothelial function in apolipoprotein E-deficient mice. J. Pharmacol. Exp. Ther. 343 (2), 253–257. 10.1124/jpet.112.194837 22837011

[B36] Lin S.-M. Liu P. P.-S. Tu Y.-K. Lai E. C.-C. Yeh J.-I. Hsu J.-Y. (2023). Risk of heart failure in elderly patients with atrial fibrillation and diabetes taking different oral anticoagulants: a nationwide cohort study. Cardiovasc. Diabetol. 22 (1), 1. 10.1186/s12933-022-01688-1 36609317 PMC9824984

[B37] Lippi G. Mattiuzzi C. Cervellin G. Favaloro E. J. (2017). Direct oral anticoagulants: analysis of worldwide use and popularity using google trends. Ann. Transl. Med. 5 (16), 322. 10.21037/atm.2017.06.65 28861419 PMC5566735

[B38] Maeda M. Tsuboi T. Hayashi T. (2019). An inhibitor of activated blood coagulation factor X shows anti-endothelial senescence and anti-atherosclerotic effects. J. Vasc. Res. 56 (4), 181–190. 10.1159/000499975 31266015

[B39] Marzilli M. (2010). Pleiotropic effects of statins: evidence for benefits beyond LDL-cholesterol lowering. Am. J. Cardiovasc. Drugs 10 (2), 3–9. 10.2165/1153644-S0-000000000-00000 21391728

[B40] Mitchell J. A. Ali F. Bailey L. Moreno L. Harrington L. S. (2008). Role of nitric oxide and prostacyclin as vasoactive hormones released by the endothelium. Exp. Physiol. 93 (1), 141–147. 10.1113/expphysiol.2007.038588 17965142

[B41] Mueck W. Stampfuss J. Kubitza D. Becka M. (2014). Clinical pharmacokinetic and pharmacodynamic profile of rivaroxaban. Clin. Pharmacokinet. 53 (1), 1–16. 10.1007/s40262-013-0100-7 23999929 PMC3889701

[B42] Muscente F. De Caterina R. (2023). The new in anticoagulation: factor XI inhibitors. Eur. Heart J. Suppl. 25, B65–B68. 10.1093/eurheartjsupp/suad070 37091652 PMC10120978

[B43] Ngo A. T. P. Jordan K. R. Mueller P. A. Hagen M. W. Reitsma S. E. Puy C. (2021). Pharmacological targeting of coagulation factor XI mitigates the development of experimental atherosclerosis in low-density lipoprotein receptor-deficient mice. J. Thromb. Haemost. 19 (4), 1001–1017. 10.1111/jth.15236 33421301 PMC8549080

[B44] Noguchi D. Kuriyama N. Hibi T. Maeda K. Shinkai T. Gyoten K. (2021). The impact of dabigatran treatment on sinusoidal protection against hepatic ischemia/reperfusion injury in mice. Liver Transpl. 27 (3), 363–384. 10.1002/lt.25929 33108682 PMC7984054

[B45] Palmirotta R. (2022). Direct oral anticoagulants (DOAC): Are we ready for a pharmacogenetic approach? J. Personalized Med. 12 (1), 17. 10.3390/jpm12010017 PMC8777772 35055332

[B46] Papadaki S. Sidiropoulou S. Moschonas I. C. Tselepis A. D. (2020). Factor Xa and thrombin induce endothelial progenitor cell activation. The effect of direct oral anticoagulants. Platelets 32 (6), 807–814. 10.1080/09537104.2020.1802413 32762584

[B47] Patel M. R. Mahaffey K. W. Garg J. Pan G. Singer D. E. Hacke W. (2011). Rivaroxaban versus warfarin in nonvalvular atrial fibrillation. N. Engl. J. Med. 365 (10), 883–891. 10.1056/NEJMoa1009638 21830957

[B48] Perzborn E. Heitmeier S. Buetehorn U. Laux V. (2014). Direct thrombin inhibitors, but not the direct factor Xa inhibitor rivaroxaban, increase tissue factor-induced hypercoagulability *in vitro* and *in vivo* . J. Thromb. Haemost. 12 (7), 1054–1065. 10.1111/jth.12591 24766850 PMC4285304

[B49] Pingel S. Tiyerili V. Mueller J. Werner N. Nickenig G. Mueller C. (2014). Thrombin inhibition by dabigatran attenuates atherosclerosis in ApoE deficient mice. Arch. Med. Sci. 10 (1), 154–160. 10.5114/aoms.2014.40742 24701228 PMC3953984

[B50] Pirmohamed M. (2006). Warfarin: almost 60 years old and still causing problems. Br. J. Clin. Pharmacol. 62 (5), 509–511. 10.1111/j.1365-2125.2006.02806.x 17061959 PMC1885167

[B51] Pistrosch F. Matschke J. B. Schipp D. Schipp B. Henkel E. Weigmann I. (2021). Rivaroxaban compared with low-dose aspirin in individuals with type 2 diabetes and high cardiovascular risk: a randomised trial to assess effects on endothelial function, platelet activation and vascular biomarkers. Diabetologia 64 (12), 2701–2712. 10.1007/s00125-021-05562-9 34495376 PMC8563606

[B52] Puech C. Delavenne X. He Z. Forest V. Mismetti P. Perek N. (2019). Direct oral anticoagulants are associated with limited damage of endothelial cells of the blood-brain barrier mediated by the thrombin/PAR-1 pathway. Brain Res. 1719, 57–63. 10.1016/j.brainres.2019.05.024 31121158

[B53] Puech C. Hodin S. Forest V. He Z. Mismetti P. Delavenne X. (2018). Assessment of HBEC-5i endothelial cell line cultivated in astrocyte conditioned medium as a human blood-brain barrier model for ABC drug transport studies. Int. J. Pharm. 551 (1-2), 281–289. 10.1016/j.ijpharm.2018.09.040 30240829

[B54] Ragia G. Manolopoulos V. G. (2022). The revolution of pharmaco-omics: ready to open new avenues in materializing precision medicine? Pharmacogenomics 23 (16), 869–872. 10.2217/pgs-2022-0145 36285650

[B55] Rahadian A. Fukuda D. Salim H. M. Yagi S. Kusunose K. Yamada H. (2020). Thrombin inhibition by dabigatran attenuates endothelial dysfunction in diabetic mice. Vasc. Pharmacol. 124, 106632. 10.1016/j.vph.2019.106632 31759113

[B56] Rajendran P. Rengarajan T. Thangavel J. Nishigaki Y. Sakthisekaran D. Sethi G. (2013). The vascular endothelium and human diseases. Int. J. Biol. Sci. 9 (10), 1057–1069. 10.7150/ijbs.7502 24250251 PMC3831119

[B57] Rogula S. Gąsecka A. Mazurek T. Navarese E. P. Szarpak Ł. Filipiak K. J. (2022). Safety and efficacy of DOACs in patients with advanced and end-stage renal disease. Int. J. Environ. Res. Public Health 19 (3), 1436. 10.3390/ijerph19031436 35162472 PMC8835601

[B58] Sanada F. Taniyama Y. Muratsu J. Otsu R. Iwabayashi M. Carracedo M. (2016). Activated factor X induces endothelial cell senescence through IGFBP-5. Sci. Rep. 6, 35580. 10.1038/srep35580 27752126 PMC5067718

[B59] Schumacher W. A. Luettgen J. M. Quan M. L. Seiffert D. A. (2010). Inhibition of factor XIa as a new approach to anticoagulation. Arteriosclerosis, Thrombosis, Vasc. Biol. 30 (3), 388–392. 10.1161/ATVBAHA.109.197178 20139363

[B60] Schwarb H. Tsakiris D. A. (2016). New direct oral anticoagulants (DOAC) and their use today. Dent. J. (Basel) 4 (1), 5. 10.3390/dj4010005 29563447 PMC5851208

[B61] Seki K. Mizuno Y. Sakashita T. Nakano S. Tanno J. Okazaki Y. (2017). Demeanor of rivaroxaban in activated/inactivated FXa. J. Pharmacol. Sci. 133 (3), 156–161. 10.1016/j.jphs.2017.02.010 28314697

[B62] Shi M. Wang L. Zhou J. Ji S. Wang N. Tong L. (2018). Direct factor Xa inhibition attenuates acute lung injury progression via modulation of the PAR-2/NF-κB signaling pathway. Am. J. Transl. Res. 10 (8), 2335–2349. 30210674 PMC6129539

[B63] Simmers M. B. Cole B. K. Ogletree M. L. Chen Z. Xu Y. Kong L.-j. (2016). Hemodynamics associated with atrial fibrillation directly alters thrombotic potential of endothelial cells. Thrombosis Res. 143, 34–39. 10.1016/j.thromres.2016.04.022 27179130

[B64] Smeda M. Stojak M. Przyborowski K. Sternak M. Suraj-Prazmowska J. Kus K. (2022). Direct thrombin inhibitor dabigatran compromises pulmonary endothelial integrity in a murine model of breast cancer metastasis to the lungs; the role of platelets and inflammation-associated haemostasis. Front. Pharmacol. 13, 834472. 10.3389/fphar.2022.834472 35295330 PMC8918823

[B84] Spronk H. M. de Jong A. M. Crijns H. J. Schotten U. Van Gelder I. C. Ten Cate H. (2014). Pleiotropic effects of factor Xa and thrombin: what to expect from novel anticoagulants. Cardiovascular res. 101 (3), 344–351. 10.1093/cvr/cvt343 24385341

[B65] Stacy Z. A. Call W. B. Hartmann A. P. Peters G. L. Richter S. K. (2016). Edoxaban: A comprehensive review of the Pharmacology and clinical data for the management of atrial fibrillation and venous thromboembolism. Cardiol. Ther. 5 (1), 1–18. 10.1007/s40119-016-0058-2 26935434 PMC4906085

[B66] Torramade-Moix S. Palomo M. Vera M. Jerez D. Moreno-Castaño A. B. Zafar M. U. (2021). Apixaban downregulates endothelial inflammatory and prothrombotic phenotype in an *in vitro* model of endothelial dysfunction in uremia. Cardiovasc Drugs Ther. 35 (3), 521–532. 10.1007/s10557-020-07010-z 32651897

[B67] van Ryn J. Goss A. Hauel N. Wienen W. Priepke H. Nar H. (2013). The discovery of dabigatran etexilate. Front. Pharmacol. 4, 12. 10.3389/fphar.2013.00012 23408233 PMC3569592

[B68] van Ryn J. Stangier J. Haertter S. Liesenfeld K. H. Wienen W. Feuring M. (2010). Dabigatran etexilate--a novel, reversible, oral direct thrombin inhibitor: interpretation of coagulation assays and reversal of anticoagulant activity. Thromb. Haemost. 103 (6), 1116–1127. 10.1160/th09-11-0758 20352166

[B69] Vianello F. Sambado L. Goss A. Fabris F. Prandoni P. (2016). Dabigatran antagonizes growth, cell-cycle progression, migration, and endothelial tube formation induced by thrombin in breast and glioblastoma cell lines. Cancer Med. 5 (10), 2886–2898. 10.1002/cam4.857 27600331 PMC5083743

[B70] Villari A. Giurdanella G. Bucolo C. Drago F. Salomone S. (2017). Apixaban enhances vasodilatation mediated by protease-activated receptor 2 in isolated rat arteries. Front. Pharmacol. 8, 480. 10.3389/fphar.2017.00480 28769809 PMC5513931

[B71] Vittal Rao H. Bihaqi S. W. Iannucci J. Sen A. Grammas P. (2021). Thrombin signaling contributes to high glucose-induced injury of human brain microvascular endothelial cells. J. Alzheimers Dis. 79 (1), 211–224. 10.3233/jad-200658 33252072

[B72] von Drygalski A. Bhat V. Gale A. J. Averell P. M. Cramer T. J. Elias D. J. (2020). An engineered factor Va prevents bleeding induced by direct-acting oral anticoagulants by different mechanisms. Blood Adv. 4 (15), 3716–3727. 10.1182/bloodadvances.2020001699 32777068 PMC7422119

[B73] Wu T. C. Chan J. S. Lee C. Y. Leu H. B. Huang P. H. Chen J. S. (2015). Rivaroxaban, a factor Xa inhibitor, improves neovascularization in the ischemic hindlimb of streptozotocin-induced diabetic mice. Cardiovasc Diabetol. 14, 81. 10.1186/s12933-015-0243-y 26077117 PMC4473833

[B74] Weitz J. I. Connolly S. J. Patel I. Salazar D. Rohatagi S. Mendell J. (2010). Randomised, parallel-group, multicentre, multinational phase 2 study comparing edoxaban, an oral factor Xa inhibitor, with warfarin for stroke prevention in patients with atrial fibrillation. Thromb. Haemost. 104 (3), 633–641. 10.1160/th10-01-0066 20694273

[B75] Wichaiyo S. Parichatikanond W. Visansirikul S. Saengklub N. Rattanavipanon W. (2023). Determination of the potential clinical benefits of small molecule factor XIa inhibitors in arterial thrombosis. ACS Pharmacol. Transl. Sci. 6 (7), 970–981. 10.1021/acsptsci.3c00052 37470020 PMC10353063

[B76] Woźniak E. Broncel M. Bukowska B. Gorzelak-Pabiś P. (2020). The protective effect of dabigatran and rivaroxaban on DNA oxidative changes in a model of vascular endothelial damage with oxidized cholesterol. Int. J. Mol. Sci. 21 (6), 1953. 10.3390/ijms21061953 32182973 PMC7139915

[B77] Wu K. K. Thiagarajan P. (1996). Role of endothelium in thrombosis and hemostasis. Annu. Rev. Med. 47, 315–331. 10.1146/annurev.med.47.1.315 8712785

[B78] Xu R. Wu F. Lan J. Duan P. (2021). Real-world comparison of direct-acting oral anticoagulants and vitamin K antagonists in chronic kidney disease: a systematic review and meta-analysis. Expert Rev. Hematol. 14 (5), 493–502. 10.1080/17474086.2021.1920012 33949923

[B79] Yang D. Shao J. Hu R. Chen H. Xie P. Liu C. (2017). Angiotensin II promotes the anticoagulant effects of rivaroxaban via angiotensin type 2 receptor signaling in mice. Sci. Rep. 7 (1), 369. 10.1038/s41598-017-00473-5 28337024 PMC5428434

[B80] Yates S. W. (2014). Interrupting anticoagulation in patients with nonvalvular atrial fibrillation. P t 39 (12), 858–880. 25516695 PMC4264672

[B81] Zekri-Nechar K. Zamorano-León J. J. Cortina-Gredilla M. López-de-Andrés A. Jiménez-García R. Navarro-Cuellar C. (2022a). Mitochondrial mitophagy protection combining rivaroxaban and aspirin in high glucose-exposed human coronary artery endothelial cell. An *in vitro* study. Diab Vasc. Dis. Res. 19 (5), 14791641221129877. 10.1177/14791641221129877 36250331 PMC9578168

[B82] Zekri-Nechar K. Zamorano-León J. J. Reche C. Giner M. López-de-Andrés A. Jiménez-García R. (2022b). Spike protein subunits of SARS-CoV-2 alter mitochondrial metabolism in human pulmonary microvascular endothelial cells: Involvement of factor Xa. Dis. Markers 2022, 1118195. 10.1155/2022/1118195 36438904 PMC9699787

[B83] Zolotoff C. Puech C. Roche F. Perek N. (2022). Effects of intermittent hypoxia with thrombin in an *in vitro* model of human brain endothelial cells and their impact on PAR-1/PAR-3 cleavage. Sci. Rep. 12 (1), 12305. 10.1038/s41598-022-15592-x 35853902 PMC9296553

